# A Glucose-Only Model to Extract Physiological Information from Postprandial Glucose Profiles in Subjects with Normal Glucose Tolerance

**DOI:** 10.1177/19322968211026978

**Published:** 2021-07-05

**Authors:** Manuel M. Eichenlaub, Natasha A. Khovanova, Mary C. Gannon, Frank Q. Nuttall, John G. Hattersley

**Affiliations:** 1 School of Engineering, University of Warwick, Coventry, UK; 2 Coventry NIHR CRF Human Metabolic Research Unit, University Hospitals Coventry and Warwickshire NHS Trust, Coventry, UK; 3 Institut für Diabetes-Technologie, Forschungs- und Entwicklungsgesellschaft mbH an der Universität Ulm, Ulm, Germany; 4 University Hospitals Coventry and Warwickshire NHS Trust, Coventry, UK; 5 Department of Medicine, Minneapolis Veterans Affairs Health Care System / University of Minnesota, Minneapolis, MN, USA

**Keywords:** glucose-only model, insulin sensitivity, glucose appearance, insulin dynamics

## Abstract

**Background:**

Current mathematical models of postprandial glucose metabolism in people with normal and impaired glucose tolerance rely on insulin measurements and are therefore not applicable in clinical practice. This research aims to develop a model that only requires glucose data for parameter estimation while also providing useful information on insulin sensitivity, insulin dynamics and the meal-related glucose appearance (GA).

**Methods:**

The proposed glucose-only model (GOM) is based on the oral minimal model (OMM) of glucose dynamics and substitutes the insulin dynamics with a novel function dependant on glucose levels and GA. A Bayesian method and glucose data from 22 subjects with normal glucose tolerance are utilised for parameter estimation. To validate the results of the GOM, a comparison to the results of the OMM, obtained by using glucose and insulin data from the same subjects is carried out.

**Results:**

The proposed GOM describes the glucose dynamics with comparable precision to the OMM with an RMSE of 5.1 ± 2.3 mg/dL and 5.3 ± 2.4 mg/dL, respectively and contains a parameter that is significantly correlated to the insulin sensitivity estimated by the OMM (*r* = 0.7) Furthermore, the dynamic properties of the time profiles of GA and insulin dynamics inferred by the GOM show high similarity to the corresponding results of the OMM.

**Conclusions:**

The proposed GOM can be used to extract useful physiological information on glucose metabolism in subjects with normal glucose tolerance. The model can be further developed for clinical applications to patients with impaired glucose tolerance under the use of continuous glucose monitoring data.

## Introduction

Mathematical models are a powerful tool to describe and assess the body’s response to food intake in people with normal glucose tolerance as well as prediabetes and type 2 diabetes mellitus (T2DM). These models typically utilise glucose and insulin data after an oral glucose intake for parameter estimation. They have contributed significantly to the understanding of the metabolic processes responsible for the loss of glycaemic control.^1-4^ Despite this success, the application of any of the proposed models in clinical practice, that is, for the diagnosis or treatment of individuals with impaired glucose tolerance, has yet to be seen. This lack of clinical application can mainly be attributed to the high cost, unreliability and dependence on venous access of insulin measurements, prohibiting widespread clinical or ambulatory insulin data collection.^5,6^ This paper thus aims to develop a glucose-only model (GOM) that describes postprandial glucose dynamics and provides physiological information while only relying on glucose data for parameter estimation.

Excluding a vast number of GOMs for type 1 diabetes mellitus,^7^ where information on exogenous insulin administration can be used during model identification, a comparatively small number of GOMs applied to subjects with normal and impaired glucose tolerance has been published. A subgroup of these GOMs is based on the description of a harmonic oscillator with an impulse input. While this significantly limits their physiological interpretation, these GOMs have been shown to contain parameters that are dependent on glucose tolerance.^8-11^ Other GOMs are based on physiological principles, but can only roughly approximate the postprandial glucose dynamics and have been applied to a very limited number of subjects.^12,13^ The main weakness of all mentioned GOMs, however, is that their results have not been validated against the results of a model known to provide accurate physiological information. Specifically, this pertains to insulin sensitivity, insulin dynamics and the meal-related appearance of glucose (GA). To overcome this weakness, this work will develop a new GOM based on and validated by the results of the oral minimal model (OMM) of glucose dynamics, identified from glucose and insulin data.^14^ The OMM has been validated by gold-standard reference methods in the past and provides an estimation of insulin sensitivity and GA.^15-17^ By identifying the novel GOM and the OMM from data of the same subjects, it is possible to validate and compare both models, particularly with respect to the GOM’s ability to provide physiological information on insulin sensitivity, insulin dynamics and GA.

## Methods

### Data Description

The dataset used in this work was collected by Ahmed et al.^18^ and Nuttall et al.^19^ and is publically accessible.^20^ It contains plasma glucose and insulin profiles from subjects with normal glucose tolerance (NGT) collected over 12 hours in a single day, where subjects consumed three identical meals four hours apart. Blood samples were collected at the same time in each subject after meal consumption at 0, 2, 5, 10, 20, 30, 40, 50, 60 min, then every 15 min up to 120 min and then every 30 min up to 240 min. One additional fasting sample was collected before breakfast, that is, at −15 min.

**Figure 1.**
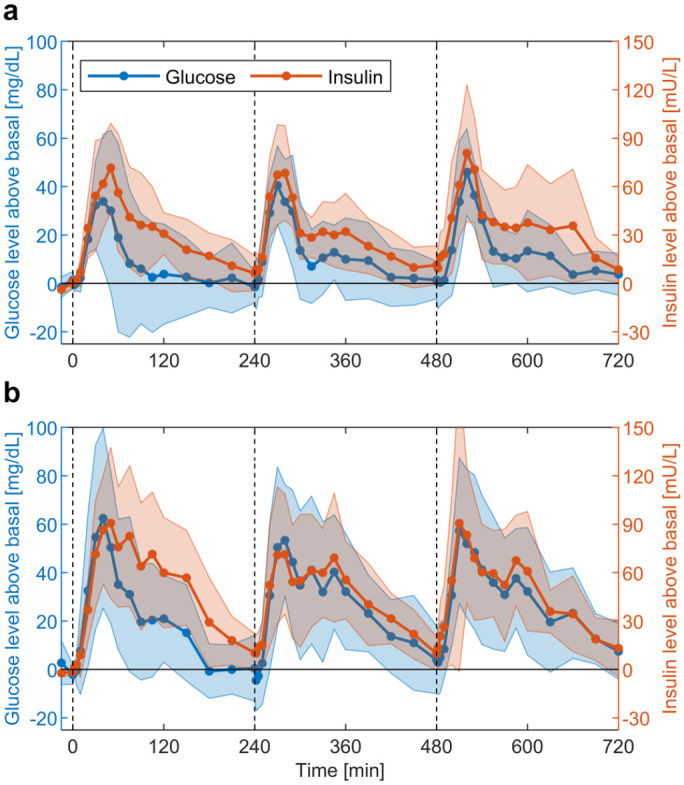
Mean and standard deviation (shaded areas) of the glucose and insulin profiles above basal levels for the two meal types of (a) standard (STAND) and (b) high carbohydrate (HCHO) composition utilised in this paper. The basal level is calculated for each subject individually as the average of the −15, 0, 2 and 5 min measurement points. The vertical dashed lines indicate the time of meal consumption.

In this work glucose and insulin profiles from 22 subjects consuming two different meal types of standard (STAND) and high carbohydrate (HCHO) macronutrient composition are used, leading to a total of 66 recorded responses. The average glucose and insulin profiles are shown in Figure 1. The absolute amount of macronutrients provided was scaled according to the body weight and female subjects received 12.5% fewer calories per body weight. Details on the subjects and consumed meals are provided in Table 1.

**Table 1.** Details on the Subject Characteristics and Different Meal Types Containing Standard (STAND) and High Carbohydrate (HCHO) Mixtures of Macronutrient Content.

	STAND	HCHO
Number of subjects (females)	12 (5)	10 (4)
Age	23 ± 1	25 ± 3
Body weight males (females) [kg]	76 ± 5 (59 ± 1)	77 ± 4 (59 ± 5)
Meal composition [% CHO/Fat/Protein]	40/49/11	63/27/10
CHO per meal (females) [g/kg body weight]	1.2 (1.1)	2 (1.8)
Calories per meal (females) [kcal/kg body weight]	13 (11)	13 (11)

The meal composition is given in percentage of calories contained in the respective macronutrient content. Data are given as mean ± standard error.

### Model Formulation

The GOM proposed in this work is based on the following generalised formulation of the OMM^14^:

(1) $$\begin{array}{l} \frac{dG\left(t\right)}{dt}=-G\left(t\right)X\left(t\right)-p_{1}\left[G\left(t\right)-G_{b}\right]\\ +\frac{Ra_{PL}\left(t\right)+Rap\left(t\right)}{V}\;\;\;\;\;\;\;\;G\left(0\right)=G_{0}, \end{array}$$

(2) $$\frac{dX\left(t\right)}{dt}=-p_{2}\left(X\left(t\right)-S_{I}\left[I\left(t\right)-I_{b}\right]\right),\;\;X\left(0\right)=X_{0},$$

**Figure 2.**
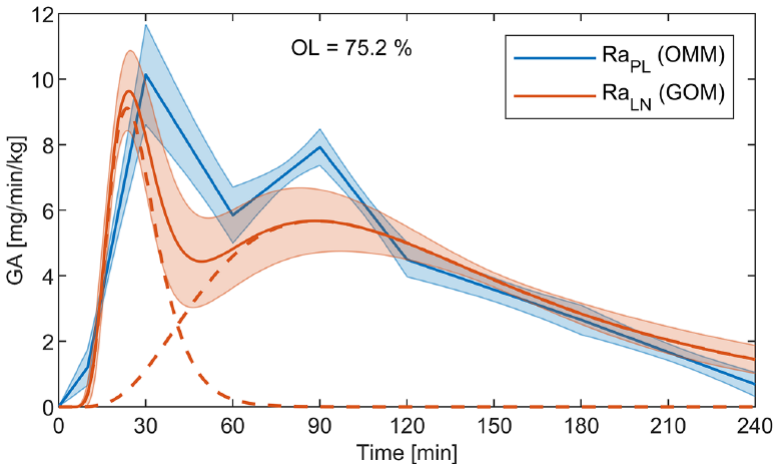
Example of the piecewise linear GA function $Ra_{PL}$ used in the oral minimal model (OMM) and the log-normally based GA function $Ra_{LN}$ used in the glucose-only model (GOM) with associated 95% confidence intervals (shaded area). The confidence intervals overlap for 75% (OL value) of the response duration of 240 min. The dashed lines indicate the two components of $Ra_{LN}$ .

The glucose concentration, its basal level and initial condition are represented by G , $G_{b}$ and $G_{0}$ (mg/dL), respectively. Parameters $p_{1}$ (min^−1^) and V (dL/kg) represent the glucose effectiveness and distribution volume of glucose relative to body weight, respectively. The state X (min^−1^) and its initial condition $X_{0}$ represent the insulin action in a remote compartment with parameter $p_{2}$ (min^−1^) governing its decay dynamics and $S_{I}$ (min^−1^ per mU/L) representing insulin sensitivity. The insulin concentration I (mU/L) and its basal level $I_{b}$ are considered to be known, error-free inputs. The input function $Ra_{PL}$ (mg/kg/min) describes the meal-related, posthepatic GA and is described by a piecewise linear function with seven breakpoints at adjustable heights and a fixed area under the curve (AUC), calculated based on the carbohydrate content of the meal (Figure 2). The function Rap represents the persisting GA originating from a previously consumed meal. The measurement process of the glucose levels is considered to be affected by an additive, normally distributed error with zero mean and a known standard deviation. The unknown parameters to be estimated from glucose and insulin data are $p_{1}$ , $p_{2}$ , $S_{I}$ and the adjustable heights of GA function $Ra_{PL}$ . The details of the model and parameter estimation procedure have been described previously.^14^

To formulate a GOM based on the OMM, it is necessary to remove the measured insulin levels, that is, I and $I_{b}$ as a known input. For that, the following model is proposed:

(3) $$\begin{array}{l} \frac{dG\left(t\right)}{dt}=-G\left(t\right)X\left(t\right)-p_{1}\left[G\left(t\right)-G_{b}\right]\\ +\frac{Ra_{LN}\left(t\right)+Rap\left(t\right)}{V}\;\;\;\;\;\;\;\;\;G\left(0\right)=G_{0}, \end{array}$$

(4) $$\frac{dX\left(t\right)}{dt}=-p_{2}\left[X\left(t\right)-S_{G}Z\left(t\right)\right],\;\;\;\;X\left(0\right)=X_{0},$$

(5) $$Z\left(t\right)=\frac{G\left(t\right)-G_{b}}{\underset{Z_{POS}}{\underset{︸}{1+\exp\left[-\alpha \left(G\left(t\right)-G_{b}\right)\right]}}}+\beta \frac{Ra_{LN}\left(t\right)}{V}$$

where the GA function $Ra_{LN}$ is defined as follows:

(6) $$\begin{array}{c} Ra_{LN}\left(t\right)=\;A\left(1-R_{H}\right)f_{LN}\left(t,T_{1},W_{1}\right)\\ +AR_{H}f_{LN}\left(t,T_{2},W_{2}\right), \end{array}$$

(7) $$f_{LN}\left(t,T,W\right)=\{\begin{array}{cc} 0 & if\;t=0\\ \frac{1}{t\;\sqrt{\pi W}}\exp\left[\frac{-{\left(\log\left(\frac{t}{T}\right)-\frac{W}{2}\right)}^{2}}{W}\right] & if\;t>0. \end{array}$$

The process of observing the glucose levels is considered to be identical to the OMM (details in section 1.2 of the supplementary information). Furthermore, the parameters $p_{1}$ , $p_{2}$ , $G_{b}$ , $G_{0}$ and V as well as the variables G and Rap from expressions (3) to (4) keep the same interpretation as in the OMM. Instead of the piecewise linear GA function $Ra_{PL}$ , the GOM features the fully differentiable function $Ra_{LN}$ in expressions (6) to (7) that is based on two overlapping components defined by a modified structure of the log-normal distribution (Figure 2). The function has a total fixed AUC of A (mg/kg) which is calculated from the carbohydrate content of the meals and has parameters $T_{1}$ (min), $T_{2}$ (min), $W_{1}$ and $W_{2}$ governing the peak times and general widths of the respective components. The parameter $R_{H}$ is restricted to the range (0,1) which ensures positivity of $Ra_{LN}$ and determines the contributions of each component to the total AUC. The function $Ra_{LN}$ has been suggested previously as a replacement for the piecewise linear function $Ra_{PL}$ in the context of the OMM, where additional details on the function can be found.^14^

The main adaptation of the GOM (3) to (7) in comparison to the OMM (1) to (2) is the introduction of the variable Z (mg/dL) in place of the insulin profile above baseline $\left[I-I_{b}\right]$ . This adaptation results in the fact that the state X (min^−1^) and its initial condition $X_{0}$ in expression (4) of the GOM no longer represent the active insulin. Instead, the state X is interpreted as a general glucose-lowering effect and the parameter $S_{G}$ (min^−1^ per mg/dL) replaces the insulin parameter $S_{I}$ in the OMM. It is thus expected that the parameter $S_{G}$ contains similar information as the insulin parameter $S_{I}$ .

The formulation of the variable Z in expression (5) is based on the general similarity between glucose and insulin dynamics, especially during the initial rise of a meal response, as demonstrated in Figure 1. This similarity allows the assumption that the information contained in the insulin data can be partially recovered from the glucose data. A similar supposition is made in several models of insulin secretion,^21-24^ where glucose levels are considered to be a known input and the primary driver of insulin secretion and therefore insulin levels. Despite the similarity between glucose and insulin dynamics, Figure 1 reveals two main differences that need to be considered by the GOM.

**Figure 3.**
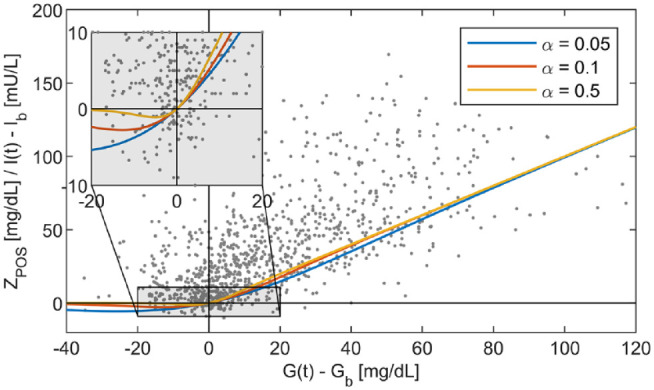
Example of the function $Z_{POS}$ for varying values of the shape parameter α . Overlayed are the simulatenously measured glucose and insulin samples above baseline to illustrate their relationship approximated by the function $Z_{POS}$ .

Firstly, it is far more prevalent for glucose levels to fall below the basal level $G_{b}$ than it is for insulin levels to fall below $I_{b}$ . This effect is incorporated by using the function $Z_{POS}$ (mg/dL) in expression (5), shown in Figure 3. The function has an approximately linear relationship to its input G(t) for $G\left(t\right)>G_{b}$ , but approaches zero for $G\left(t\right)<G_{b}$ , with the parameter α (dL/mg) governing the shape of the transition (Figure 3). Secondly, the average glucose and insulin profiles above baseline in Figure 1 indicate that, after a simultaneous rise, insulin levels often remain elevated for longer and decay slower toward the baseline levels in comparison to glucose levels. This is especially prominent in all responses to the STAND meal and after breakfast in the HCHO meal. The GOM accommodates this behaviour by including the GA function $Ra_{LN}$ in the description of Z . This allows the variable Z to remain elevated even when glucose levels have reached basal levels and the contribution of $Z_{POS}$ vanishes. The parameter β (min) acts as a unit conversion factor and adjusts the strength of the coupling between $Ra_{LN}$ and the variable Z . A comparable feature is also included in the previously mentioned models of insulin secretion,^22-24^ where the rate of change of glucose levels, which is highly dependent on $Ra_{LN}$ as indicated by expression (3), is thought to affect insulin secretion and therefore its levels.

### Parameter Estimation

The dataset contains three consecutive meal responses from each subject that are considered separately during parameter estimation in the GOM, that is, one set of unknown parameters is estimated from every meal response. To incorporate the overlapping effects of consecutive meals, the parameter estimation procedure previously described for the OMM is utilised.^14^ The procedure adapts the initial conditions of the states, $G_{0}$ and $X_{0}$ , as well as the persisting GA Rap based on the time of meal consumption, while keeping the basal level of glucose $G_{b}$ constant throughout the day (details in section 1.1 of the supplementary information).

The following parameters of the GOM (3) to (7) are considered for estimation: system parameters $p_{1}$ , $p_{2}$ , $S_{G}$ and β , and parameters $T_{1}$ , $T_{2}$ , $W_{1}$ , $W_{2}$ and $R_{H}$ governing the log-normally based GA function $Ra_{LN}$ . Using the observability rank criterion,^25,26^ it can be shown that these parameters are structurally locally identifiable (details in section 1.3 of the supplementary information). The shape parameter α of $Z_{POS}$ is fixed to a value of 0.1 dL/mg as a stochastic sensitivity analysis revealed that it is practically unidentifiable, that is, it cannot be estimated with an acceptable level of precision (details in section 1.4 of the supplementary information). The value of 0.1 dL/mg is chosen as it approximates the relationship between glucose and insulin data suitably (Figure 3).

The parameter estimation is carried out using a variational Bayesian approach,^27-29^ which has been used previously to identify low dimensional models including the OMM.^10,14,30-32^ This approach provides a probabilistic treatment of unknown parameters which allows the estimation of parameter uncertainty and requires the specification of prior distributions over unknown parameters. All unknown parameters of the GOM are specified as log-normally distributed and characterised by their median and coefficient of variation (CV) since the parameters are only physiologically plausible when positive. The exception to that is the parameter $R_{H}$ which is restricted to the range (0,1). The details of the chosen prior distributions are provided in section 1.4 of the supplementary information. For the parameters $p_{1}$ and $p_{2}$ as well as the GA function parameters, the same prior distributions as in the OMM are used.^14^ For the newly introduced parameters $S_{G}$ and β , a stochastic sensitivity analysis was carried out to ensure that the chosen prior distributions can capture the variabitly of response from the data (details in section 1.4 of the supplementary information). Here, it should be mentioned that due to the chosen GOM formulation, parameters β and $S_{G}$ show significant covariance, which leads to poor estimation precision when both parameters have wide prior distributions. Due to the importance of the parameter $S_{G}$ for carrying information on insulin sensitivity, its prior CV was chosen to be 50%, while simultaneously using a narrow prior distribution (CV of 10 %) for the parameter β .

### Validation

The validity of the results produced by the GOM is assessed by comparing the corresponding results of the OMM obtained with the identical approach from the same dataset.^14^ In particular, the following aspects are compared between the OMM and the GOM:

- Model fit as assessed through the time profile of residuals between the model-inferred and observed glucose levels, weighted by the measurement error and the root mean squared residuals (RMSE).
- Information on insulin sensitivity as assessed through correlation and comparison between the parameter $S_{G}$ of the GOM and parameter $S_{I}$ of the OMM.
- Agreement of the inferred time profiles of GA, that is, the piecewise-linear GA function $Ra_{PL}$ of the OMM and the log-normally based GA function $Ra_{LN}$ . To quantify this agreement for every response, the confidence interval (CI) associated with the individual GA profiles is inferred from the posterior distributions of the unknown parameters of $Ra_{PL}$ and $Ra_{LN}$ . Subsequently, the time during which the 95 % CIs of $Ra_{PL}$ and $Ra_{LN}$ overlap (OL) is calculated and expressed as the share of the total response time of 240 min (Figure 2). High OL percentages thus indicate high similarity between $Ra_{PL}$ and $Ra_{LN}$ .
- Agreement of the time course of insulin dynamics represented by $S_{I}\left[I\left(t\right)-I_{b}\right]$ in expression (2) for the OMM, abbreviated as $Y_{OMM}$ , and inferred by $S_{G}Z\left(t\right)$ in expression (4) by the GOM, abbreviated as $Y_{GOM}$ . As these quantities enter the description of the state X at the same position in both OMM and GOM, $Y_{GOM}$ could carry information on insulin dynamics. Analogous to the GA profiles, the agreement of $Y_{OMM}$ and $Y_{GOM}$ is quantified as the share of time during with the 95 % CIs overlap. To calculate the CI for $Y_{OMM}$ , the reported insulin assay CV of 13 % is used.^18^

## Results

**Figure 4.**
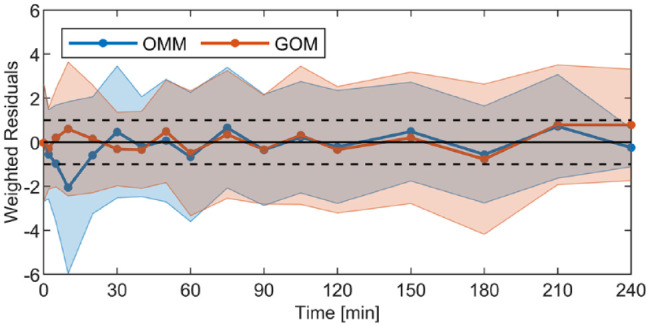
Mean and standard deviation of weighted residuals between the model-inferred and observed glucose levels for the oral minimal model (OMM) and the glucose-only model (GOM) identified on the same dataset.

The parameter estimation of the GOM was carried out in all 66 recorded responses (22 subjects with 3 responses each), and the individual results are provided in section 2.1 of the supplementary information. The time profile of weighted residuals between model-inferred and observed glucose levels is displayed in Figure 4. These results demonstrate that the model is capable of describing the glucose data well, as all average weighted residuals are contained within the −1/+1 range. Additionally, it is demonstrated that in comparison to the OMM results, the GOM shows a smaller error in the first 30 min of the responses. The RMSE values of the GOM are statistically equivalent to the RMSE values of the OMM, that is, 5.1 ± 2.3 mg/dL for the GOM and 5.3 ± 2.4 mg/dL for the GOM (*P* = .73).

**Figure 5.**
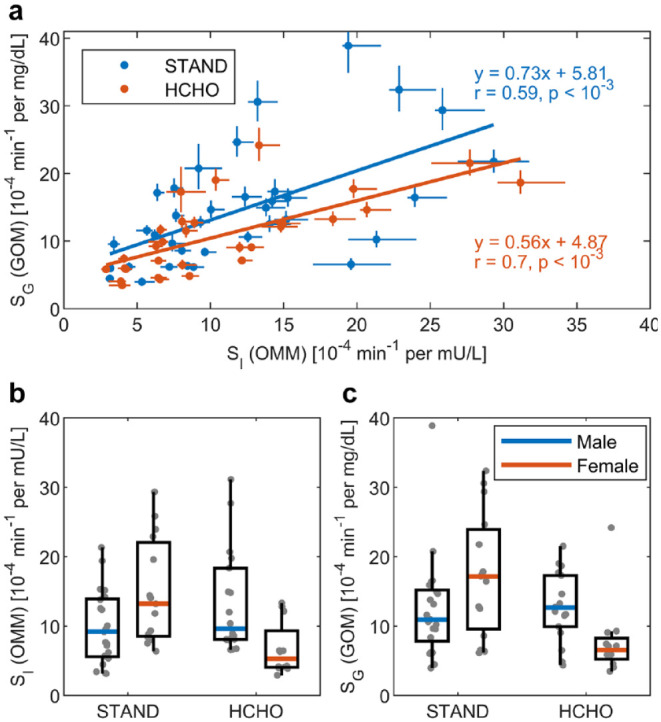
Results of the parameter $S_{G}$ from glucose-only model (GOM) and the corresponding insulin sensitivity parameter $S_{I}$ from oral minimal model (OMM). The results are compared between the meals of standard (STAND) and high carbohydrate (HCHO) composition. Plot (a) shows a correlation and linear regression analysis with the horizontal and vertical lines for each data point indicating the one-sigma range of the posterior log-normal parameter distribution. Plots (b, c) show boxplots of $S_{I}$ and $S_{G}$ separated by meal type and subject sex.

The comparison between parameter $S_{G}$ of the GOM and parameter $S_{I}$ of the OMM are displayed in Figure 5. Firstly, the parameter $S_{G}$ can be estimated with good precision as indicated by a small posterior CV of 9.0 ± 2.5 % which is comparable to the posterior CV of $S_{I}$ (6.8 ± 5.1 %). The values of $S_{G}$ and $S_{I}$ are significantly correlated with *r* = 0.59 and *r* = 0.7 for the STAND and HCHO meals, respectively (Figure 5a). The posterior results of all parameters are provided in section 2.2 of the supplementary material.

**Figure 6.**
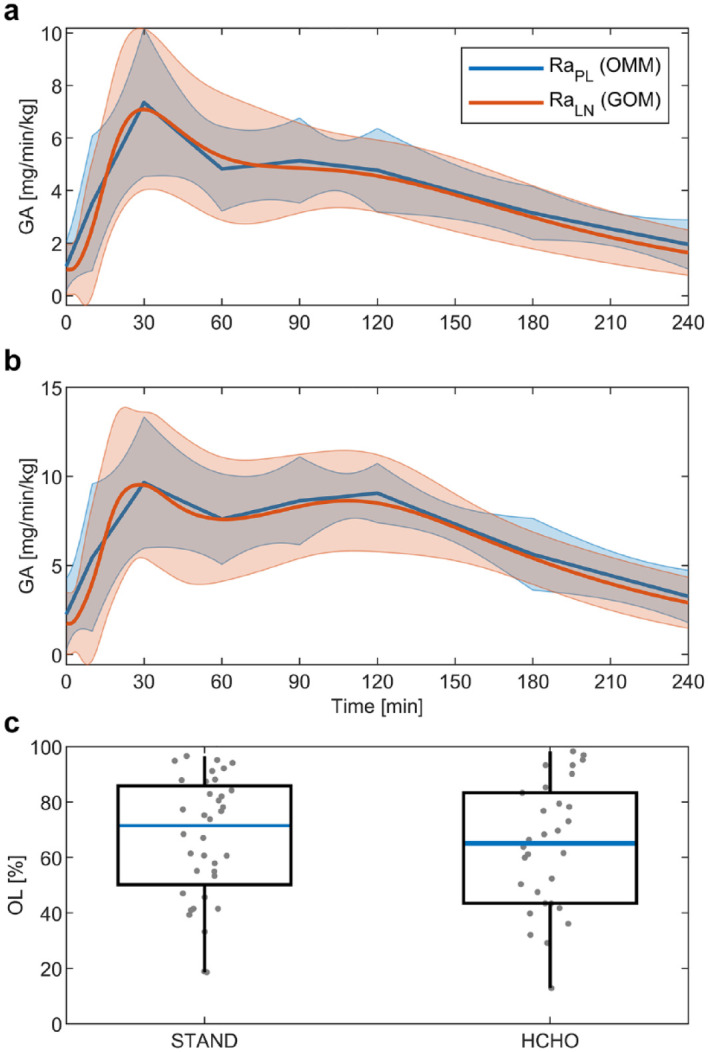
Comparison between the glucose appearance (GA) profiles estimated by the piecewise linear function $Ra_{PL}$ in the oral minimal model (OMM) and the log-normally based function $Ra_{LN}$ in the glucose-only model (GOM). Plot (a) displays to the results from the meal of standard composition (STAND) and plot (b) to the meal of high carbohydrate composition (HCHO). The results are given as mean (solid line) and standard deviation (shaded area) of all responses. Plot (c) gives boxplots of the share of time during which the 95% confidence intervals (CIs) of $Ra_{PL}$ and $Ra_{LN}$ overlap (OL values).

GA profiles from the OMM ( $Ra_{PL}$ ) and the GOM ( $Ra_{LN}$ ) are presented in Figure 6. The average profiles in plots (a) and (b) display similar dynamic properties, that is, the shoulder of GA in the STAND meal and secondary peak in the HCHO meal are correctly inferred by the GOM. There is, however, a larger difference in the first 30 min of the response due to the different mathematical formulations of the GA functions. The distribution of OL values in Figure 6c indicates no difference between meal types (*P* = .63) and shows that a majority of OL values lie above 65 %, indicating a good agreement between inferred GA profiles.

**Figure 7.**
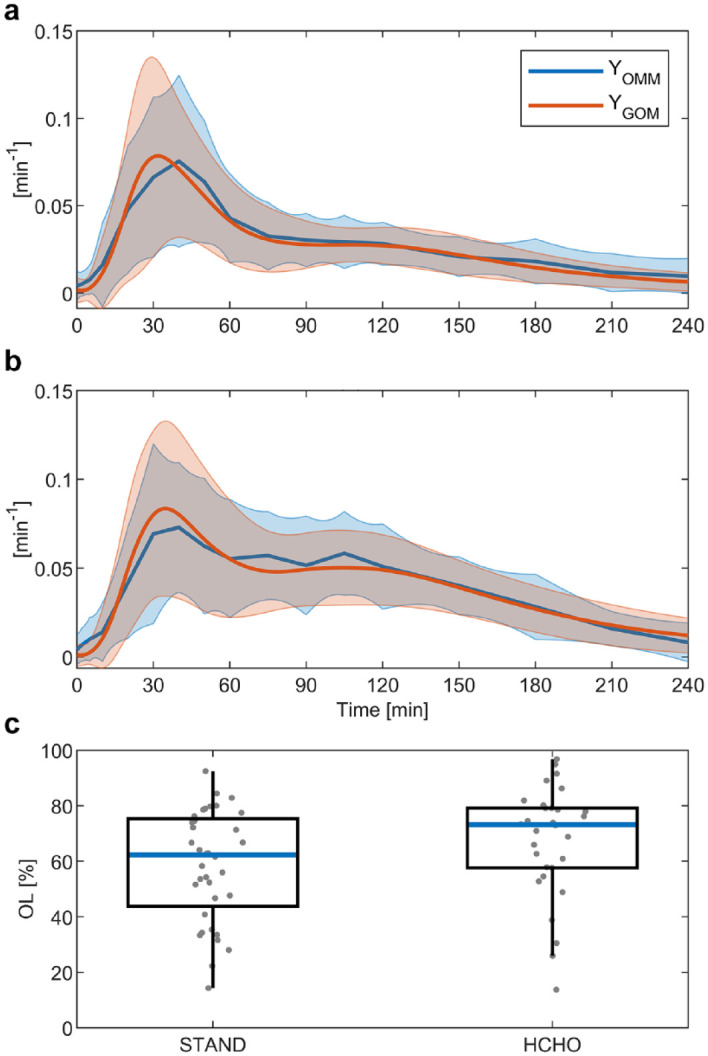
Comparison between the estimated profiles of $Y_{OMM}=S_{I}\left[I\left(t\right)-I_{b}\right]$ in the oral minimal model (OMM) and $Y_{GOM}=S_{G}Z\left(t\right)$ in the glucose-only model (GOM). Plot (a) shows to the results from the meal of standard composition (STAND) and plot (b) to the meal of high carbohydrate composition (HCHO). The results are given as mean (solid line) and standard deviation (shaded area) of all responses. Plot (c) gives boxplots of the share of time during which the 95% confidences intervals (CIs) of $Y_{OMM}$ and $Y_{GOM}$ overlap (OL values).

Analogous to the GA profiles, the time courses of $Y_{OMM}$ and $Y_{GOM}$ are compared in Figure 7. The average profiles in plots (a) and (b) show similar dynamic properties, e.g. a shoulder after the initial rise in the case of the HCHO meal. This agreement is confirmed by the distribution of OL values in Figure 7c. Of note is that the OL values of the HCHO meal are increased in comparison to the STAND meal (*P* = .08), which could be connected to the increased correlation between $S_{I}$ and $S_{G}$ estimates in the HCHO meal.

## Discussion

A glucose-based model to describe postprandial glucose responses from different meals in subjects with NGT is presented. This new GOM has been formulated and validated using the physiology-based OMM. Analysing the weighted residuals (Figure 4) and RMSE, it can be concluded that the GOM can describe the glucose data equally well and possess sufficient flexibility to account for the large variability in the responses (Figure 1).

The ability of the GOM to provide information on insulin sensitivity is indicated by a significant correlation between the parameters $S_{G}$ and $S_{I}$ (Figure 5a). Especially for the HCHO meal, the correlation coefficient of 0.7 is comparable to commonly used surrogate indices of insulin sensitivity such as HOMA-IR and the Matsuda index, where correlation values of 0.65 and 0.73, respectively, against clamping results have been reported.^33^ Further evidence for the informative value of the parameter $S_{G}$ is given by the fact that it displayes the same differences between male and female subjects for the different meal types, as the parameter $S_{I}$ (Figure 5b and 5c). In contrast, the interpretability of $S_{G}$ values across meal types is weakened by the fact that there is a significant difference between meal types (*P* = .04) that is not observed in $S_{I}$ values (*P* = .45).

There are two inherent limitations in the approach to using only glucose data to assess insulin sensitivity. Firstly, it could be rarely the case that, the dynamic properties of glucose and insulin levels, e.g. the timing and existence of peaks, can exhibit very little similarity, thus violating one of the modeling assumptions. The second limitation stems from the fact that absolute levels of insulin are not always correlated to absolute glucose levels, even when the dynamical properties of both signals are identical. This means that two subjects could have quantitatively similar glucose profiles but exhibit vastly different absolute insulin levels and thus also have different insulin sensitivities. Detecting this difference using glucose data alone is thus an inherent limitation.

In terms of GA, the results show that average profiles inferred by the GOM and OMM show very similar dynamic properties, with a larger difference in the first 30 min of the response (Figure 6). As the weighted residuals of the GOM are closer to zero in that same period (Figure 4), a more realistic estimation of GA with the log-normally based function $Ra_{LN}$ and the GOM during this period is indicated. A very similar observation was made when $Ra_{LN}$ was used in conjunction with the OMM.^14^

Similar to GA, the GOM’s ability to infer information on insulin dynamics is demonstrated by the similarity of average profiles of $Y_{OMM}$ and $Y_{GOM}$ (Figure 7). To assess the agreement between the individual results of GA and insulin dynamics, the OL value was introduced. The results of both GA and insulin dynamics indicate a satisfactory agreement but also exhibit high variability between responses (see Figures 6c and 7c as well as the individual results in the supplementary information). This variability in OL values reflects the variability in overall glucose and insulin responses (Figure 1). The interpretability of GOM results is thus less reliable on an individual level.

A general weakness of the current study is the use of a dataset that only contains subjects with NGT. To assess the model’s applicability in patients with prediabetes and T2DM, further validation and adaptation with appropriate datasets, e.g. from Peter et al.^34^ is required.

While the dataset used in this research contained glucose data from blood sampling collected in a controlled clinical setting, it would also be possible to identify the proposed GOM from more easily obtainable, ambulatory datasets. For instance, glucose profiles recorded with continuous glucose monitoring (CGM) at home, where meals are typically consumed at irregular intervals and contain varying amounts of carbohydrates, could be used. An application of the GOM to these types of datasets is in part possible as the GOM features the differentiable input function $Ra_{LN}$ that is independent of the considered response duration and easily adaptable to meals with greatly varying carbohydrate content.

## Conclusion

This paper, for the first time, proposed a glucose-based model for the successful extraction of useful physiological information on glucose metabolism in subjects with NGT, thereby overcoming the weaknesses of existing GOM approaches.^8-13^ The model’s independence from insulin measurements and exclusive use of easily accessible data enable further developments and its potential application in research and clinical practice to a large number of subjects. In particular, the proposed model could allow a more sophisticated physiological interpretation of CGM profiles collected under ambulatory conditions. It could thus support the design of personalised dietary interventions in prediabetes and T2DM or examine the glycaemic derangement in gestational diabetes mellitus.

## Supplemental Material

sj-docx-1-dst-10.1177_19322968211026978 – Supplemental material for A Glucose-Only Model to Extract Physiological Information from Postprandial Glucose Profiles in Subjects with Normal Glucose Tolerance Click here for additional data file. Supplemental material, sj-docx-1-dst-10.1177_19322968211026978 for A Glucose-Only Model to Extract Physiological Information from Postprandial Glucose Profiles in Subjects with Normal Glucose Tolerance by Manuel M. Eichenlaub, Natasha A. Khovanova, Mary C. Gannon, Frank Q. Nuttall and John G. Hattersley in Journal of Diabetes Science and Technology
