# Supplementary material for: A Glucose-Only Model to Extract Physiological Information from Postprandial Glucose Profiles in Subjects with Normal Glucose Tolerance
Source: J Diabetes Sci Technol. 2021 Jul 5;16(6):1532–40. doi: 10.1177/19322968211026978 (PMC9631515; doi:10.1177/19322968211026978)
Supplement: sj-docx-1-dst-10.1177_19322968211026978 – Supplemental material for A Glucose-Only Model to Extract Physiological Information from Postprandial Glucose Profiles in Subjects with Normal Glucose Tolerance [file sj-docx-1-dst-10.1177_19322968211026978.docx]

**Supplementary material**

Supplementary material for the article “A glucose-only model to extract physiological information from postprandial glucose profiles in subjects with normal glucose tolerance”, by Eichenlaub, Khovanova, Nuttall, Gannon and Hattersley.

# Parameter estimation

## Procedure

The procedure for incorporating the overlapping effect between meals is illustrated in Figure S1 and is very similar to the previous work by Eichenlaub et al. ^1^, where the oral minimal model was identified with the same dataset. Firstly, the baseline value of glucose $G_{b}$ is calculated from the -15, 0, 2 and 5 min glucose measurements at breakfast and fixed over the entire duration covered by the dataset (12 hours). A recalculation of the glucose basal level before every meal is unfeasible because it cannot be assumed that basal levels are reached before the next meal is consumed. In contrast, the initial condition $G_{0}$ is reset for every meal, where $G_{0}$ is calculated as the average of the 0, 2 and 5 min samples (see Figure S1). A similar approach to $X_{0}$ is not possible because this state is not directly observed but inferred by the model. Instead, $X_{0}$ is set to 0 before breakfast, assuming no active glucose-lowering effect due to the fasting state of the subjects. For the subsequent meals (lunch and dinner) this assumption cannot be justified, so $X_{0}$ is set to the last inferred value from the previous meal, i.e. $X(240)$. The persisting absorption $Rap$ is calculated as $Rap\left( t \right)={Ra}_{LN}(t+240)$, ${Ra}_{LN}$ is the glucose appearance inferred from the previous meal. For breakfast we have $Rap\left( t \right)=0$.


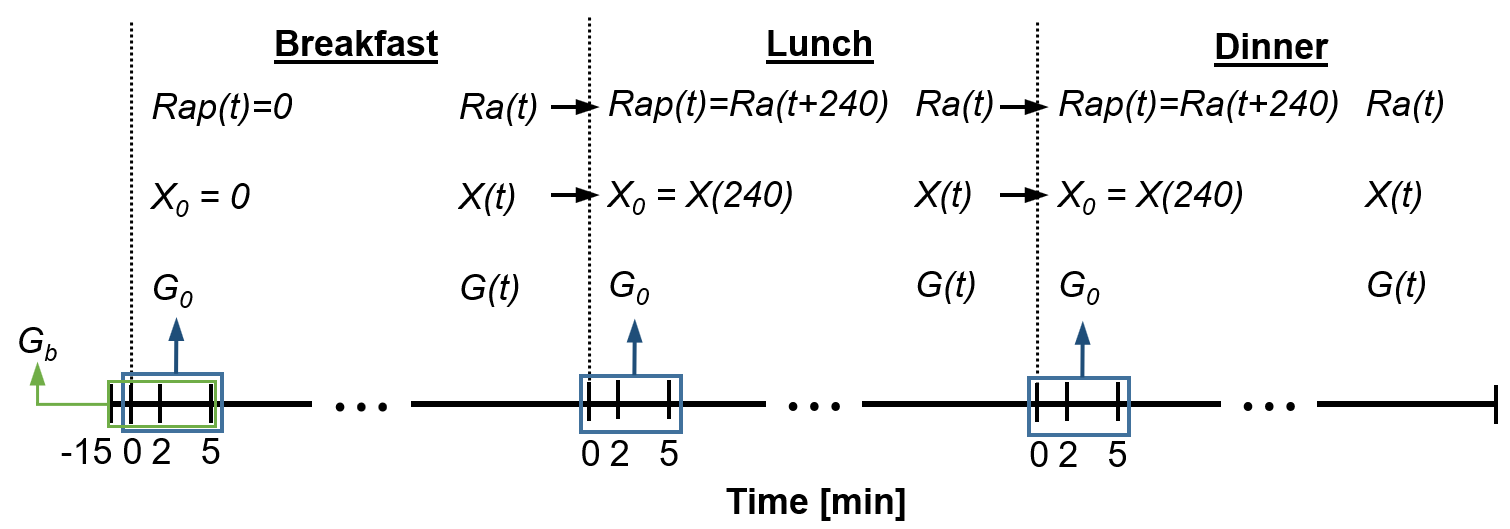


Figure S1: Schematic depiction of the procedure for incorporating the overlapping effect between meals

The fixed AUC of ${Ra}_{LN}$ ($A$) is calculated as the product of the amount of glucose in the meal per kg of body weight $D$ and the fraction $f$ of ingested glucose that enters the peripheral circulation fixed to 0.9. The distribution volume $V$ is fixed to a population average of 1.45 dL/kg.

## Measurement uncertainty

The glucose measurement process is modelled as follows

| $y\left( t \right)=G\left( t \right)+\varepsilon$ with $\varepsilon\sim\mathcal{N}\left( 0,\lambda^{2} \right),$ |  |
| --- | --- |

where $y\left( t \right)$ is the observed plasma glucose concentration and $\varepsilon$ is the normally distributed, additive measurement error with zero mean and standard deviation $\lambda$. The variational Bayesian (VB) method for parameter estimation models the measurement uncertainty with a precision parameter $\kappa=1/\lambda^{2}$. This parameter $\kappa$ is described with a Gamma distribution characterised by shape and rate parameters $a$ and $b$. Furthermore, the VB method gives the option to fix the PDF over the precision $\kappa$ during parameter estimation. In this work, we make use of this option and fix the PDF over $\kappa$ according to the known glucose assay CV of 2 %. This is done using the procedure introduced by Eichenlaub ^2^, where all details are described. In short, we start by defining the fixed distribution over the standard deviation $\lambda$ in terms of its mean and coefficient of variation. This is done for every response individually, where the CV of $\lambda$ is fixed to 10 % and the mean is set as the average glucose level of the response multiplied by the glucose assay CV. Using these values for the distribution over $\lambda$, the corresponding Gamma distribution over $\kappa$ is specified by calculating parameters $a$ and $b$ using the procedure by Eichenlaub ^2^.

To account for a change in measurement uncertainty dependant on the glucose level at each time point, the VB toolbox allows the user to specify a scaling factor of the measurement precision at each time point. These scaling factors are calculated by diving each glucose measurement point by the average glucose of the entire response.

## Structural identifiability

The observability rank criterion (ORC) method used in this work is implemented in the MATLAB toolbox STRIKE-GOLDD v2.2 (<https://sites.google.com/site/strikegolddtoolbox/>). It is based on a property of the model observability, which determines whether the model's internal states can be ascertained from the output measurements in finite time. If the parameters are thereby treated as state variables with zero dynamics, it is possible to determine their local identifiability by calculating the rank of a generalised observability-identifiability matrix using results from differential geometry. The disadvantage of this method is that it is not possible to determine the locally identifiable parameter combinations.

The GOM (3) - (7) is defined as follows

| % States  syms G X  x = [G; X];  % Inputs  syms t;  u = [t];  % Unknown parameters  syms p1 p2 SG beta T1 W1 T2 W2 Rh  p =[p1; p2; SG; beta; T1; W1; T2; W2; Rh];  % Output/observation  h = G;  % Initial Conditions  syms G0 X0  ics = [G0; X0];  known_ics = [1,1];  % Model equations  Ra_LN = (1-Rh)/(t*sqrt(pi*W1))*exp(-(log(t/T1) - W1/2)^2/W1) + ...  Rh/(t*sqrt(pi*W2))*exp(-(log(t/T2) - W2/2)^2/W2);  f = [-X*G - p1*G + Ra_LN + exp(-t);  -p2*(X-SG*(G/(1+exp(-G)) + beta*Ra_LN))]; |
| --- |

Here, we assume time to be a known input. As the toolbox does not allow the definition of known parameters, we had to assume specific values for known parameters, i.e. $G_{b}=0$, $V=1$, $Rap=\exp(-t)$, $\alpha=1$ and $A=1$. The result is that all parameters are structurally locally identifiable, as demonstrated by the following output:

| --------------------------------  >>> STRIKE-GOLDD toolbox v2.2  --------------------------------  Analyzing identifiability of GOM_ZPOS_Ra2 ...  >>> The model contains:  2 states:  [G; X]  1 outputs:  G  1 known inputs:  t  0 unknown inputs:    9 parameters:  [p1; p2; SG; beta; T1; W1; T2; W2; Rh]  >>> Building the observability-identifiability matrix requires at least 10 Lie derivatives  Calculating derivatives: 1 2 3 4 5 6 7 8 9 10  >>> Observability-Identifiability matrix built with 10 Lie derivatives  (calculated in 1.189662e+03 seconds)  >>> Calculating rank...  Rank = 11 (calculated in 4.818197e+03 seconds)  ------------------------  >>> RESULTS SUMMARY:  ------------------------  >>> The model is Fully Input-State-Parameter Observable (FISPO):  All its states are observable.  All its parameters are locally structurally identifiable.  Total execution time: 6.010907e+03 |
| --- |

## Stochastic sensitivity analysis

The results of the stochastic sensitivity analysis of the shape parameter $\alpha$ in Figure S2 show that this parameter has only a very small influence on the model output and can thus not be estimated with acceptable precision making it practically unidentifiable.


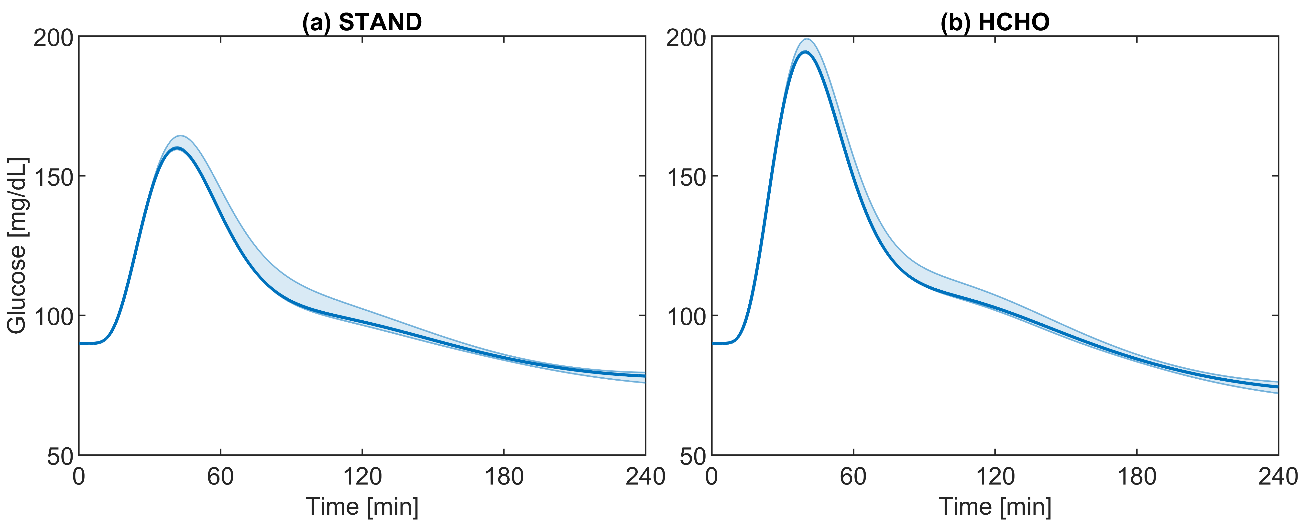


Figure S2: Results of a stochastic sensitivity analysis using a log-normal distribution of $\alpha$ with a median of 0.1 dL/mg and a CV of 100 %. The solid line gives the median and the shaded area the 95 % CI of 1000 Monte-Carlo simulations with all other parameters fixed to their prior values.

The results of the stochastic sensitivity analysis of all parameters are shown in Figure S3. For that we have drawn 1000 random Monte Carlo samples from the prior distributions and simulated the corresponding model responses. The results show that the range of model outputs covers the variability in measured glucose responses.


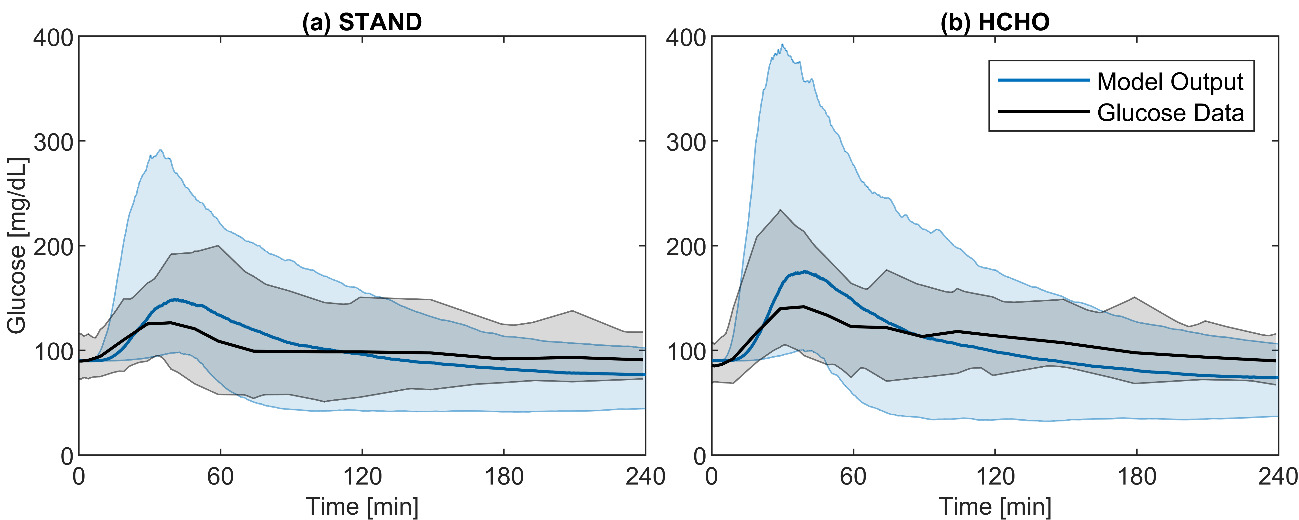


Figure S3: Results of a stochastic sensitivity analysis using the prior distributions of all parameters. The solid line gives the median and the shaded area the 95 % CI of 1000 Monte-Carlo simulations. The same holds for the data. Note that $G_{0}$ and $G_{b}$ were kept constant at 90 mg/dl for every simulation.

## Prior distributions

A summary of all prior distributions and constants used during model inversion can be found in Table S1.

Table S1: Details of prior distributions and constants used for model inversion.

| **Parameter** | **Unit** | **Prior**  **median ± CV %** | **Description** |
| --- | --- | --- | --- |
| $p_{1}$ | min^-1^ | 0.025 ± 25 ^3^ | Glucose effectiveness, log-normally distributed |
| $p_{2}$ | min^-1^ | 0.012 ± 40 ^3^ | Dynamic parameter of the state $X$, log-normally distributed |
| $S_{G}$ | 10^-4^ min^-1^ per mg/dL | 10 ± 50 | Coupling parameter between $Z$ and $X$, log-normally distributed |
| $\alpha$ | dL/mg | 0.1 (fixed) | Shape parameter of $Z_{POS}$ |
| $\beta$ | min | 5 ± 10 | Coupling parameter between ${Ra}_{LN}$ and $Z$, log-normally distributed |
| $T_{1}$, $T_{2}$ | min | [30, 100] ± 30 ^1^ | Peak times of the two components of ${Ra}_{LN}$, log-normally distributed |
| $W_{1}$, $W_{2}$ | - | 0.5 ± 30 ^1^ | Widths of the two components of ${Ra}_{LN}$, log-normally distributed |
| $R_{H}$ | - | 0.7 ± 30 ^1^ | Contributions of the AUCs of individual components to the overall AUC of ${Ra}_{LN}$, logistically distributed |
| $V$ | dL/kg | 1.45 (fixed) ^3^ | Distribution volume of glucose |
| $\lambda$ | mg/dL | (fixed) | Standard deviation of measurement error calculated from glucose assay CV of 2 % and scaled according to the glucose measurements at each time point |
| $f$ | - | 0.9 (fixed) ^3^ | Fraction of ingested glucose that is absorbed |
| $D$ | mg/kg | (fixed) | Amount of ingested glucose in the meal per kg of body weight |
| $A$ | mg/kg | (fixed) | AUC of the function ${Ra}_{LN}$ calculated as $D\cdot f$ |
| $G_{b}$ | mg/dL | (fixed) | Basal level of glucose calculated as the average of -15, 0, 2 and 5 min samples before breakfast. |
| $G_{0}$ | mg/dL | (fixed) | Initial condition of glucose calculated as the average of 0, 2 and 5 min samples before every meal of the day. |
| $X_{0}$ | min^-1^ | (fixed) | Initial condition of state X. Set to zero in the fasted state or inferred from the previous meal, i.e. X(240) |

# Additional results

## Individual results

The results of individual model fits and inferred time profiles of GA and Y are displayed in Figures S4-S14. The discontinuities between inferred glucose profiles of consecutive meals can be explained by the recalculation of $G_{0}$ based on the data as explained in section 1.1. A resetting of $G_{0}$ based on inferred previous response to ensure a smooth transition was intentionally omitted in order to avoid the transition of errors in the glucose inference from one response to the next. The discontinuities in $Y_{GOM}=S_{G}Z(t)$ have a similar cause, additional to the fact that the GA dependent term of $Z(t)$ in expression (5) of the main text, excludes the persisting absorption $Rap$.


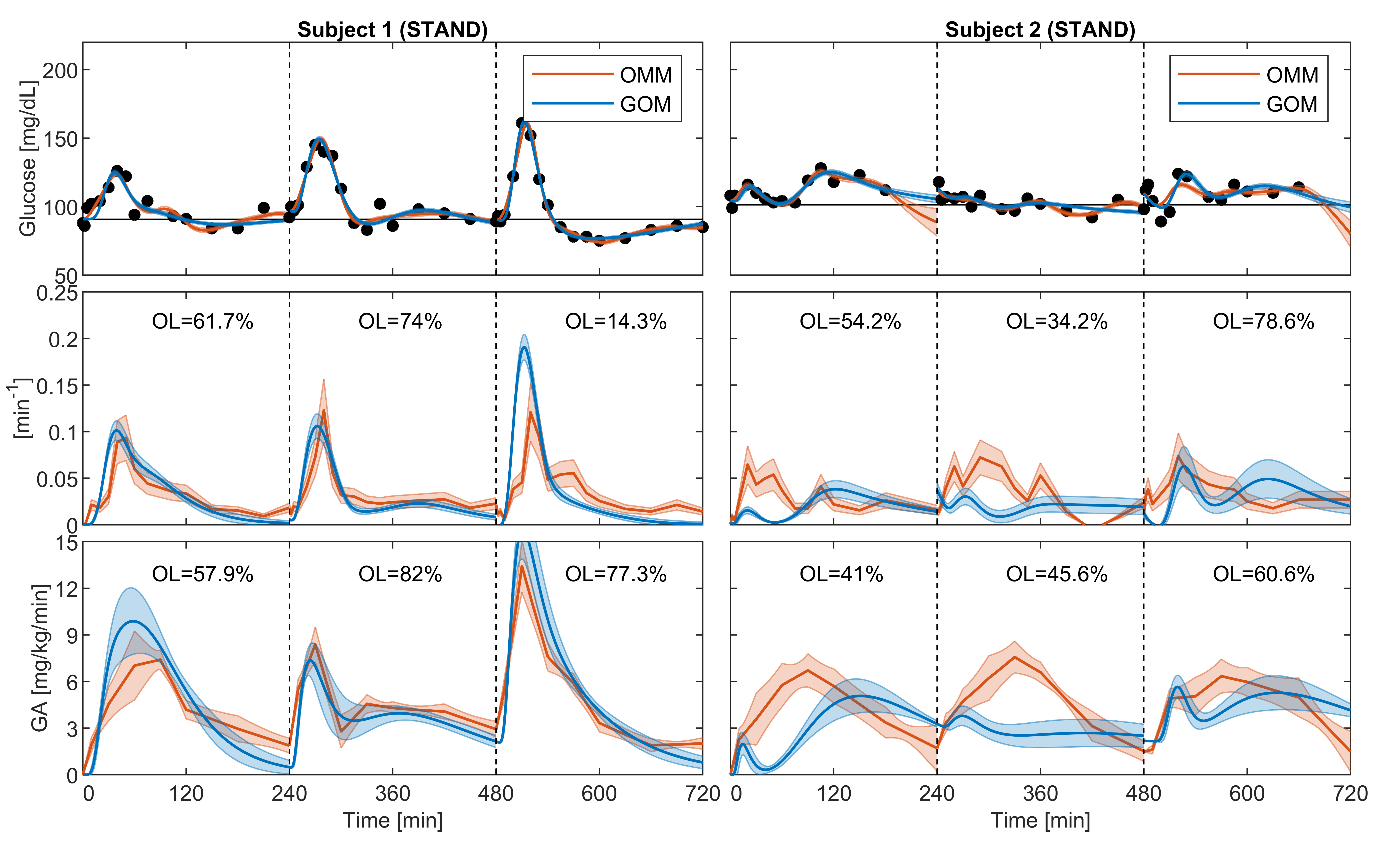


Figure S4: Comparison of the individual results of the model fit for the OMM and GOM. Shown are the data and predicted glucose profiles in the top row, the insulin dynamics in the form of $Y_{OMM}=S_{I}\left[ I\left( t \right)-I_{b} \right]$ and $Y_{GOM}=S_{G}Z(t)$ with 95 % CI in the middle row and the GA time profiles with 95 % CI in the bottom row. The dashed vertical lines indicate the time of meal consumption.

**
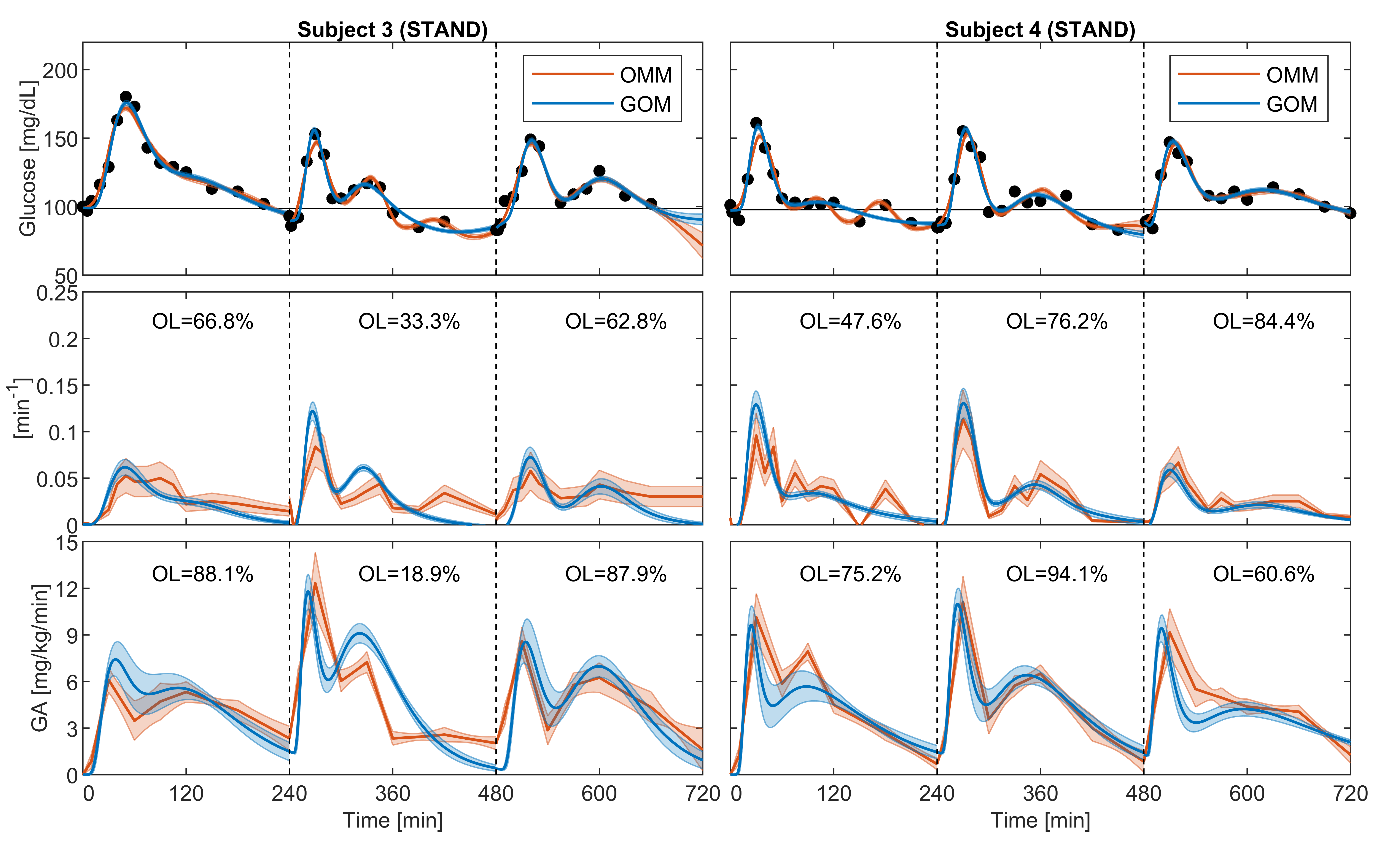
**

Figure S5: See caption of Figure S4.


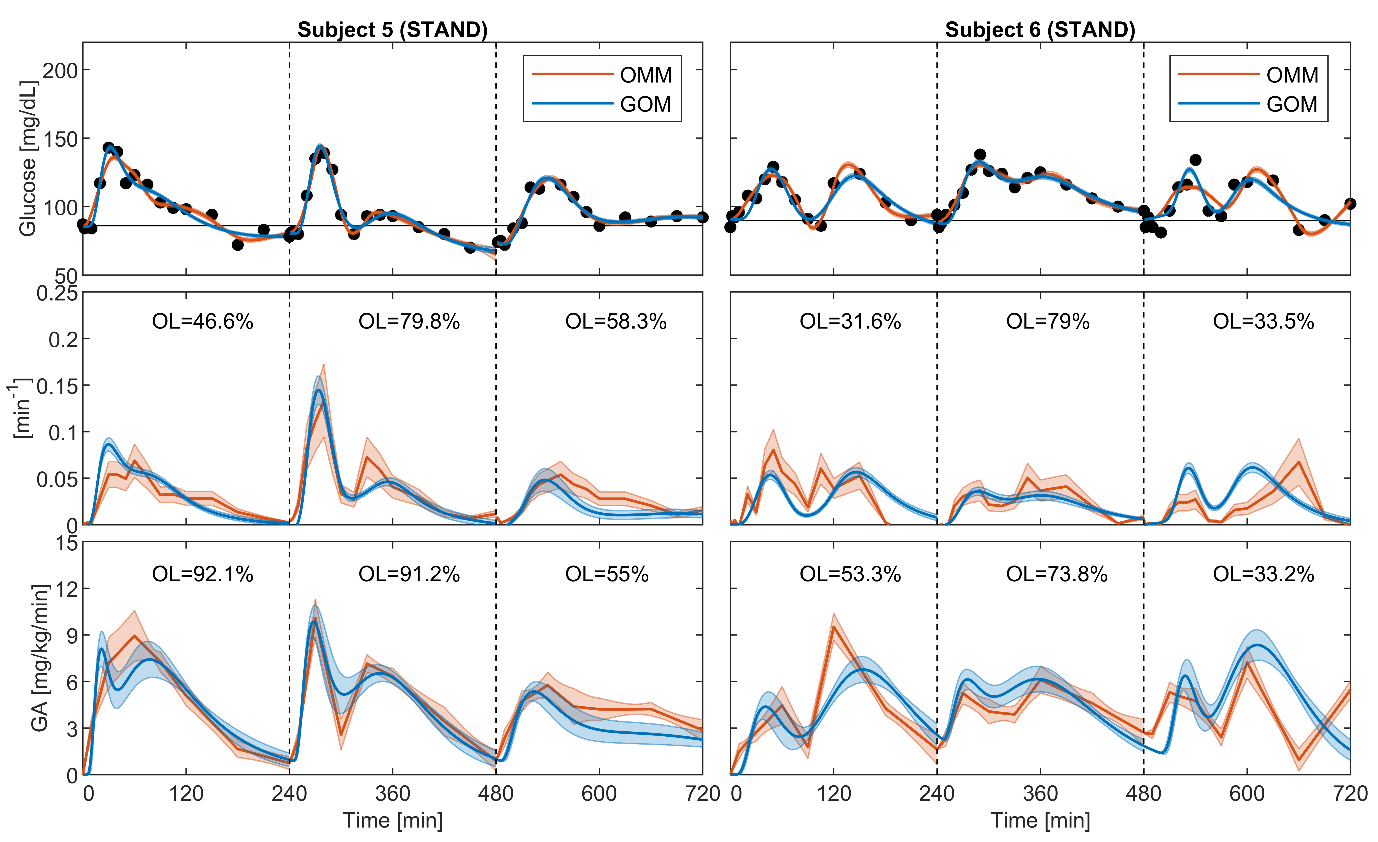


Figure S6: See caption of Figure S4.


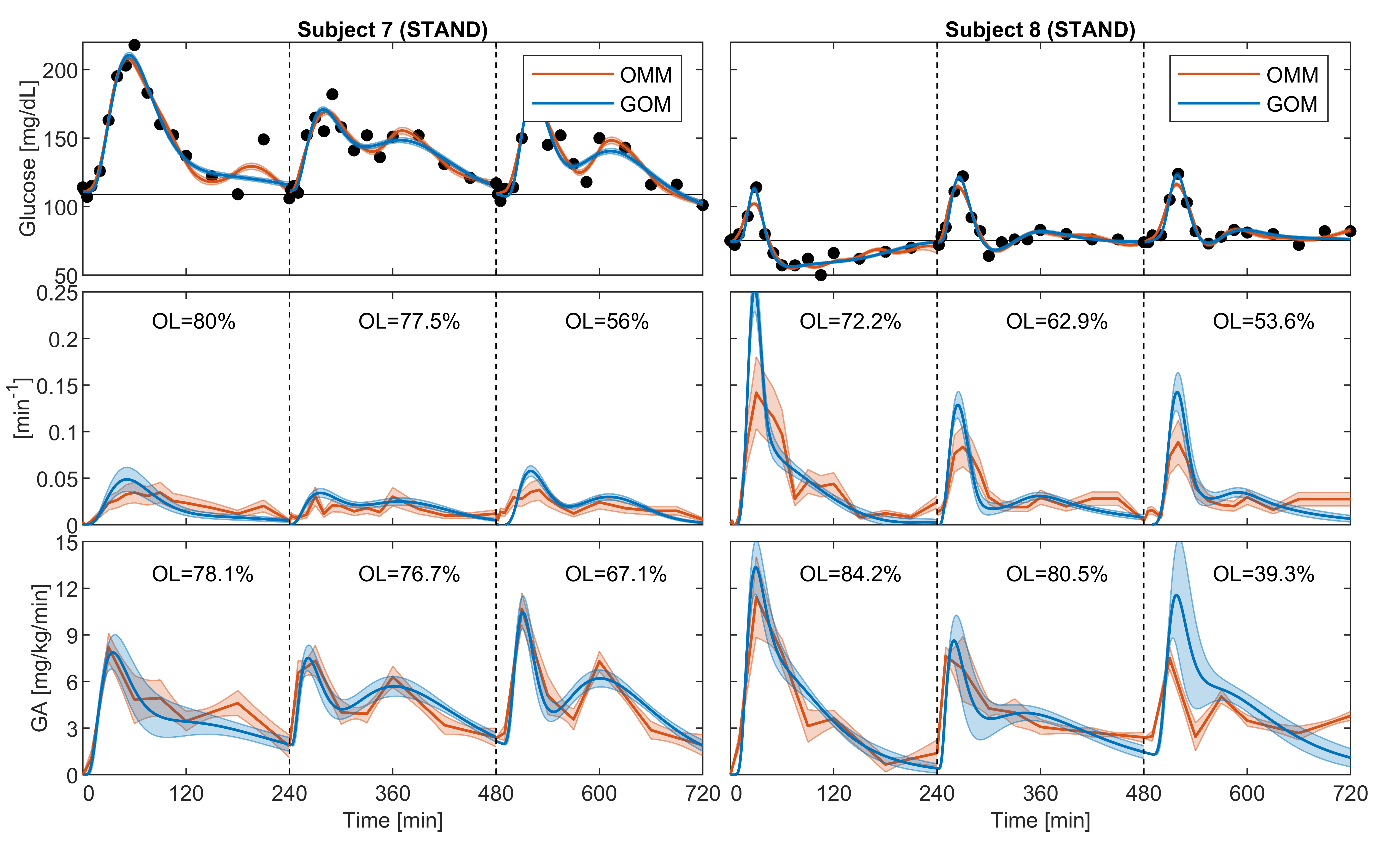


Figure S7: See caption of Figure S4.


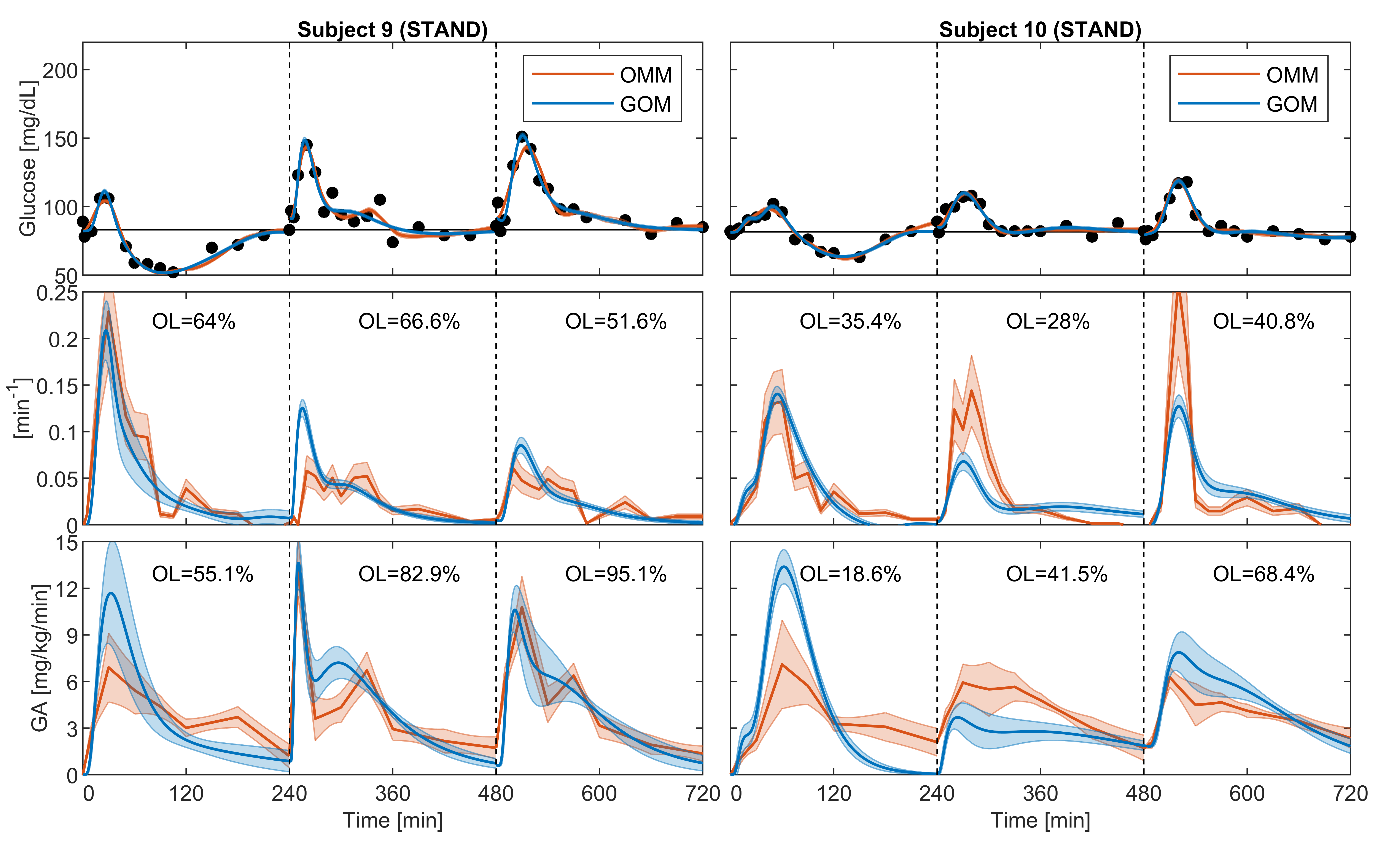


Figure S8: See caption of Figure S4.


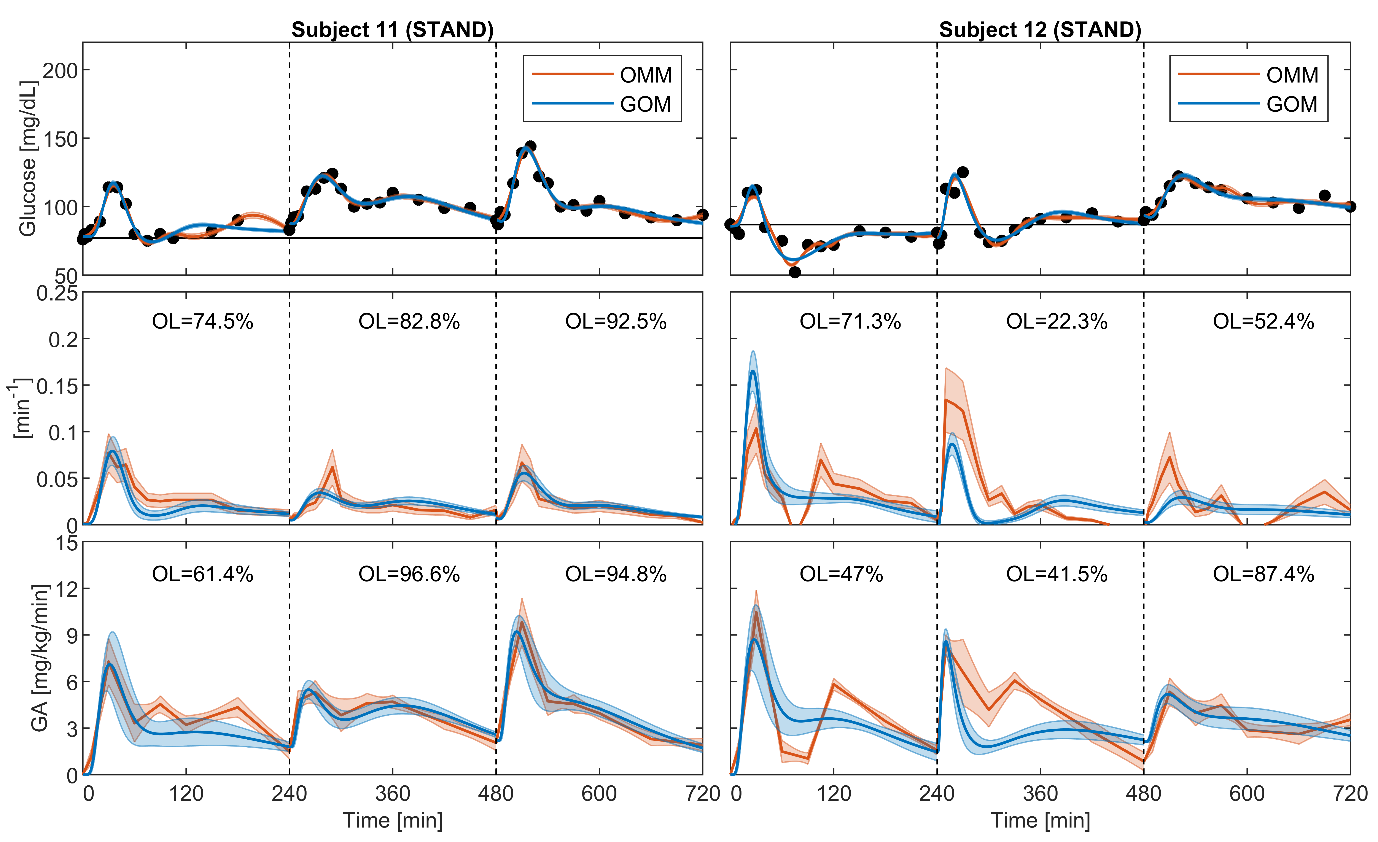


Figure S9: See caption of Figure S4.


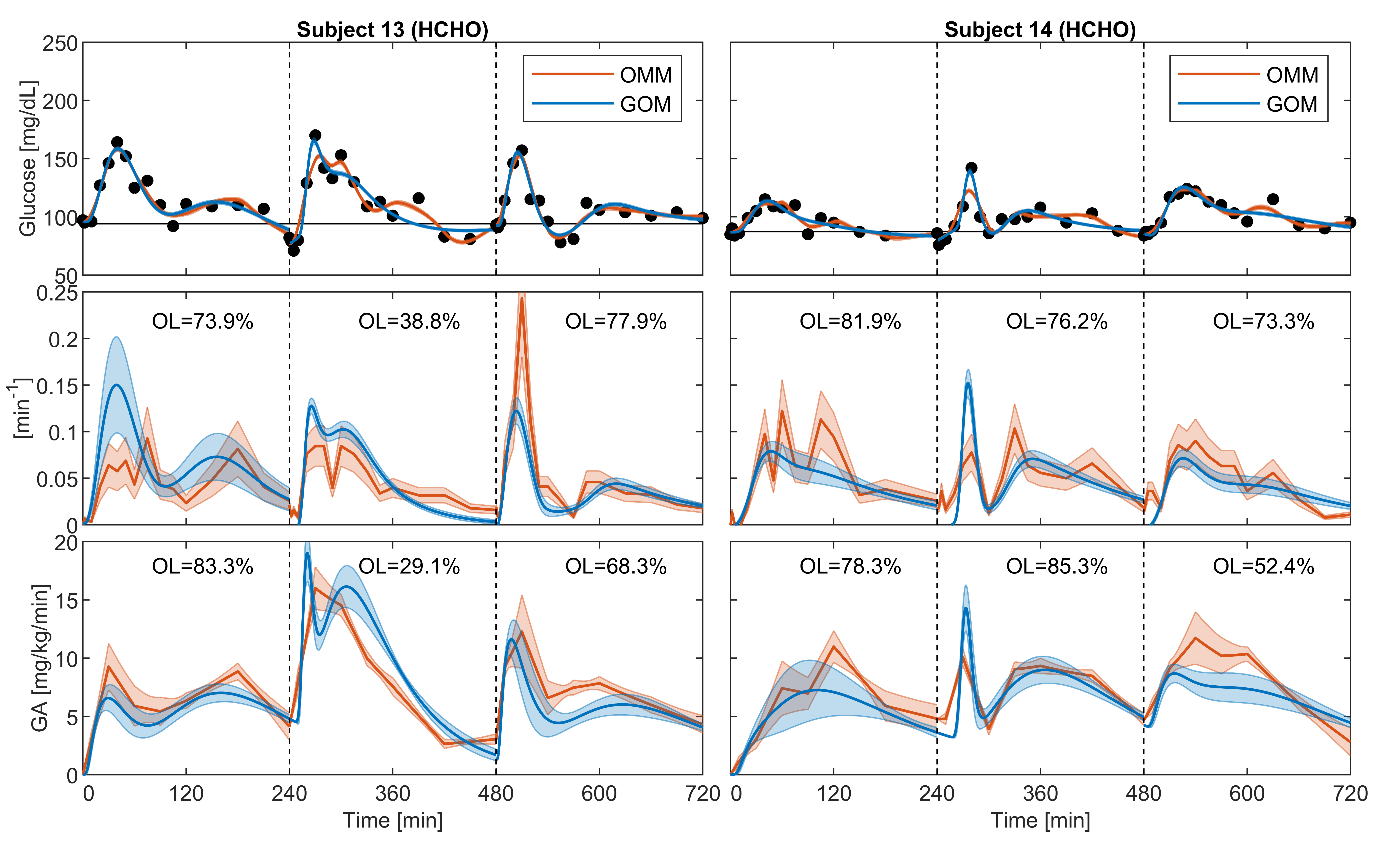


Figure S10: See caption of Figure S4.


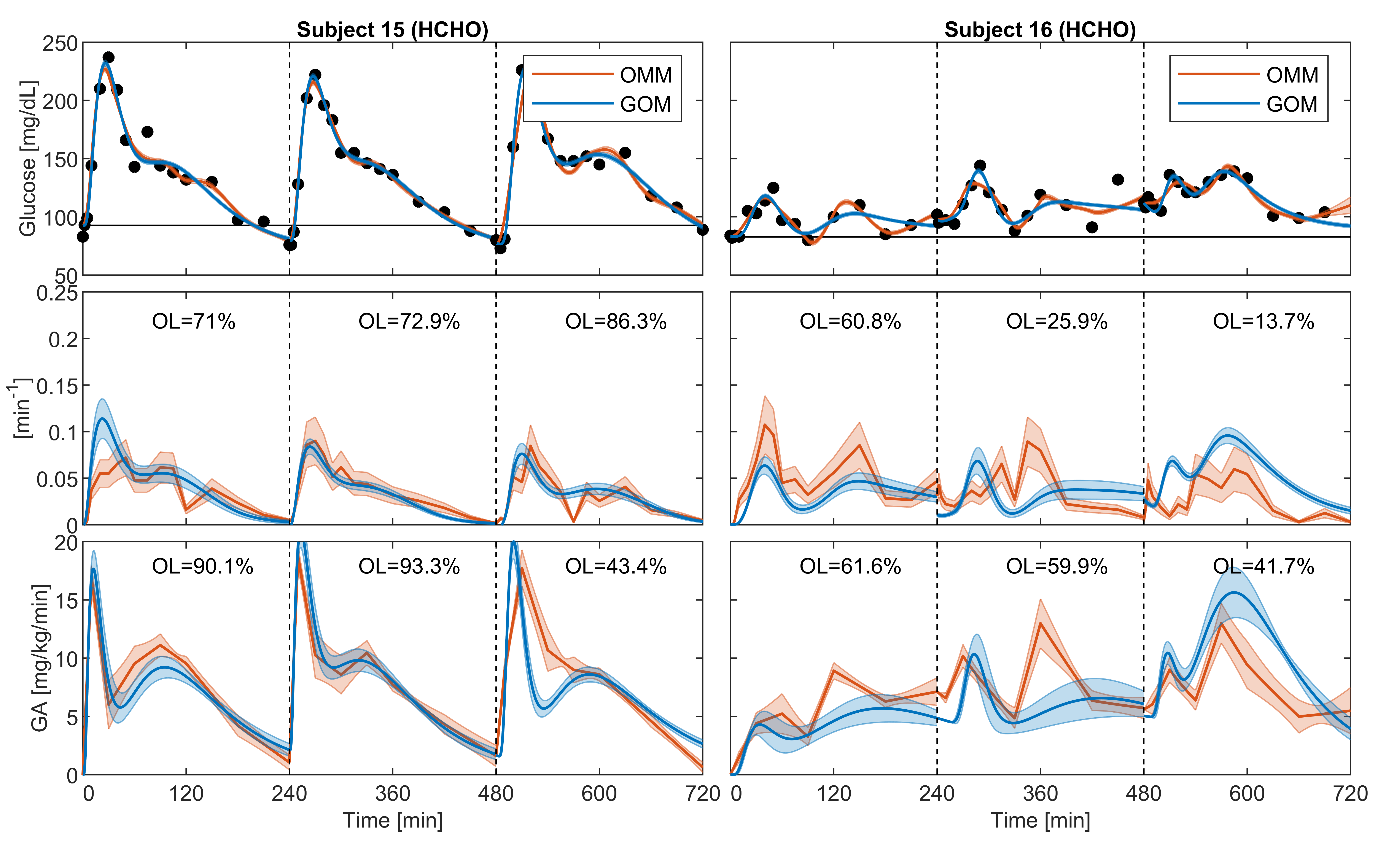


Figure S11: See caption of Figure S4.


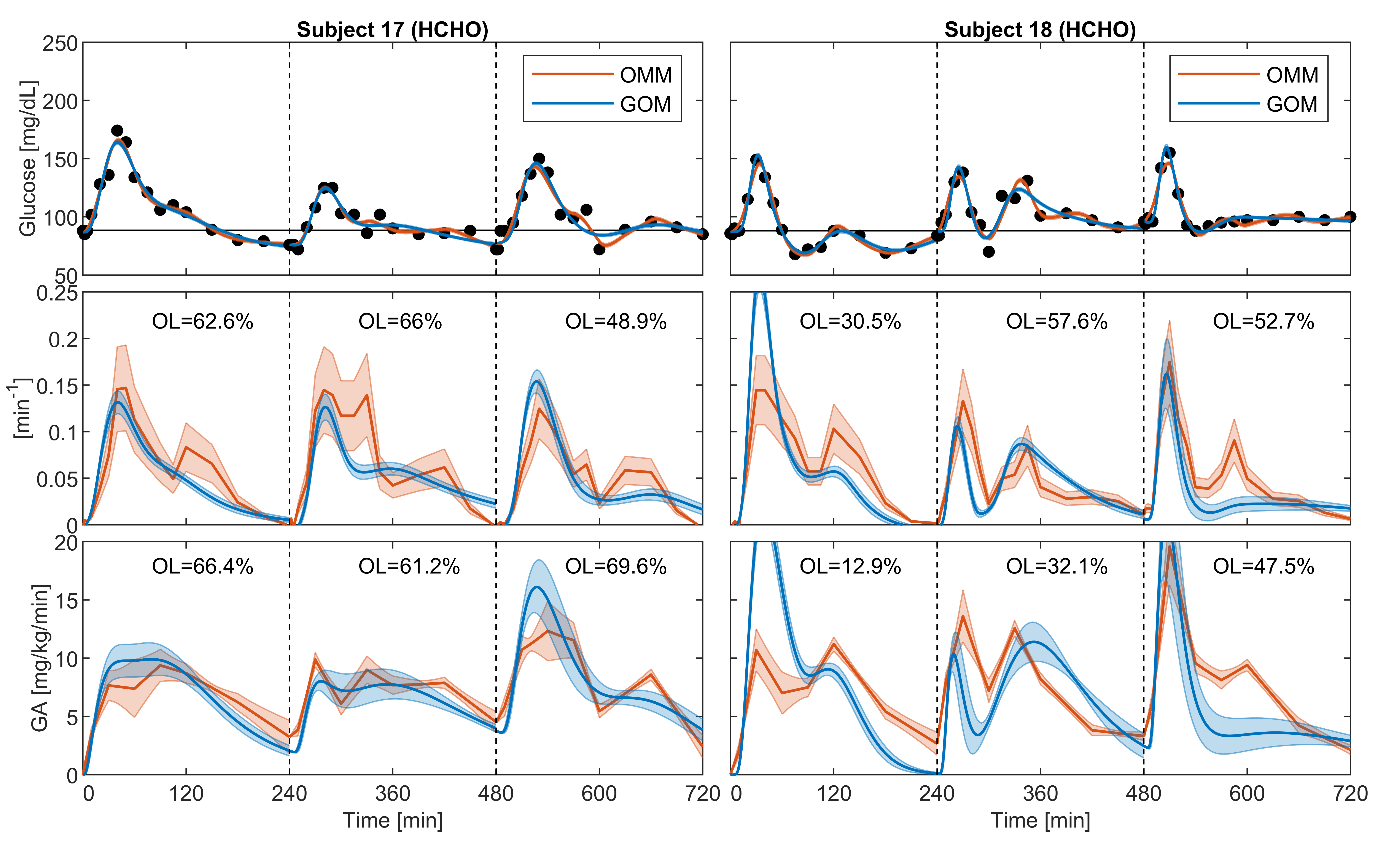


Figure S12: See caption of Figure S4.


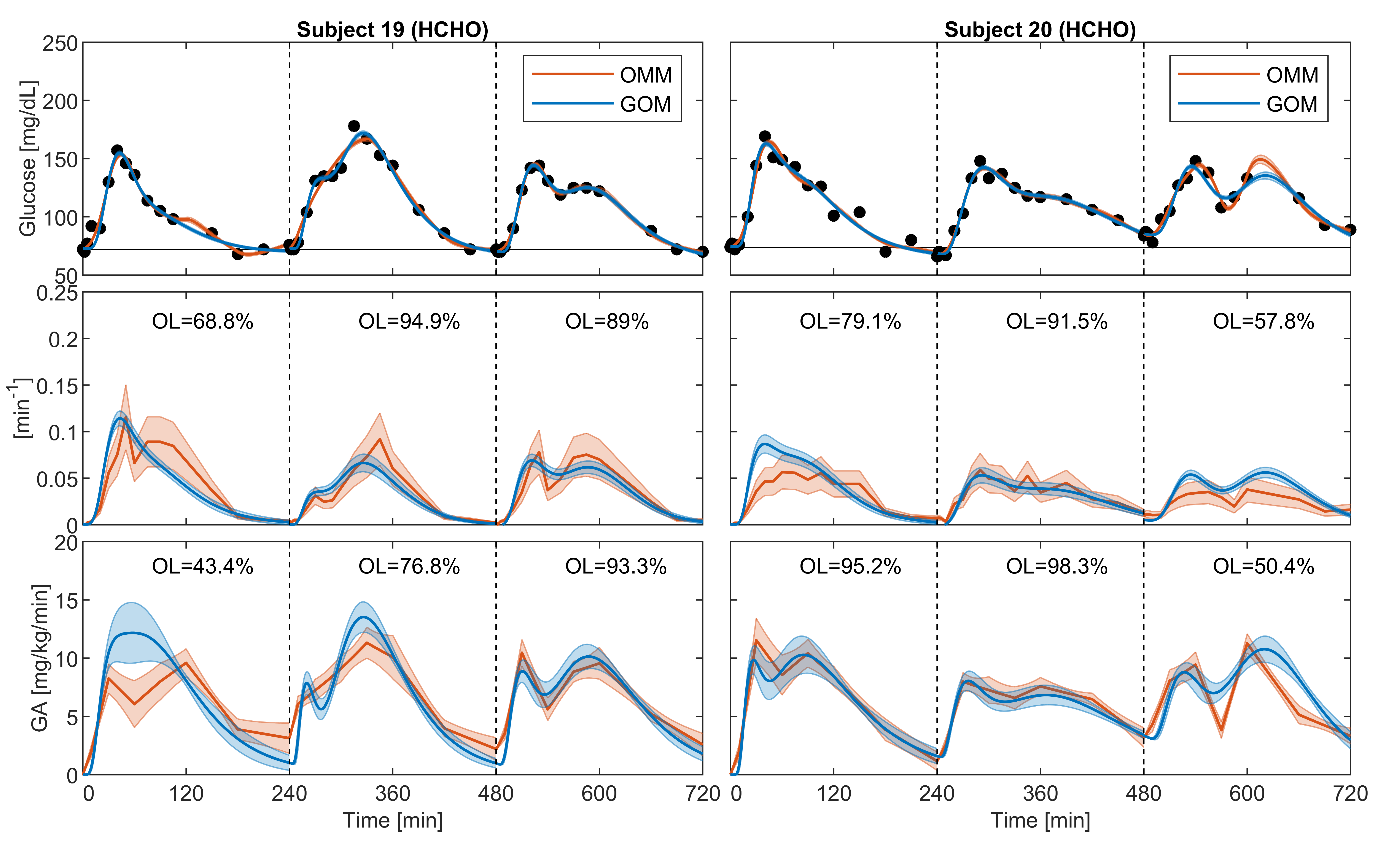


Figure S13: See caption of Figure S4.


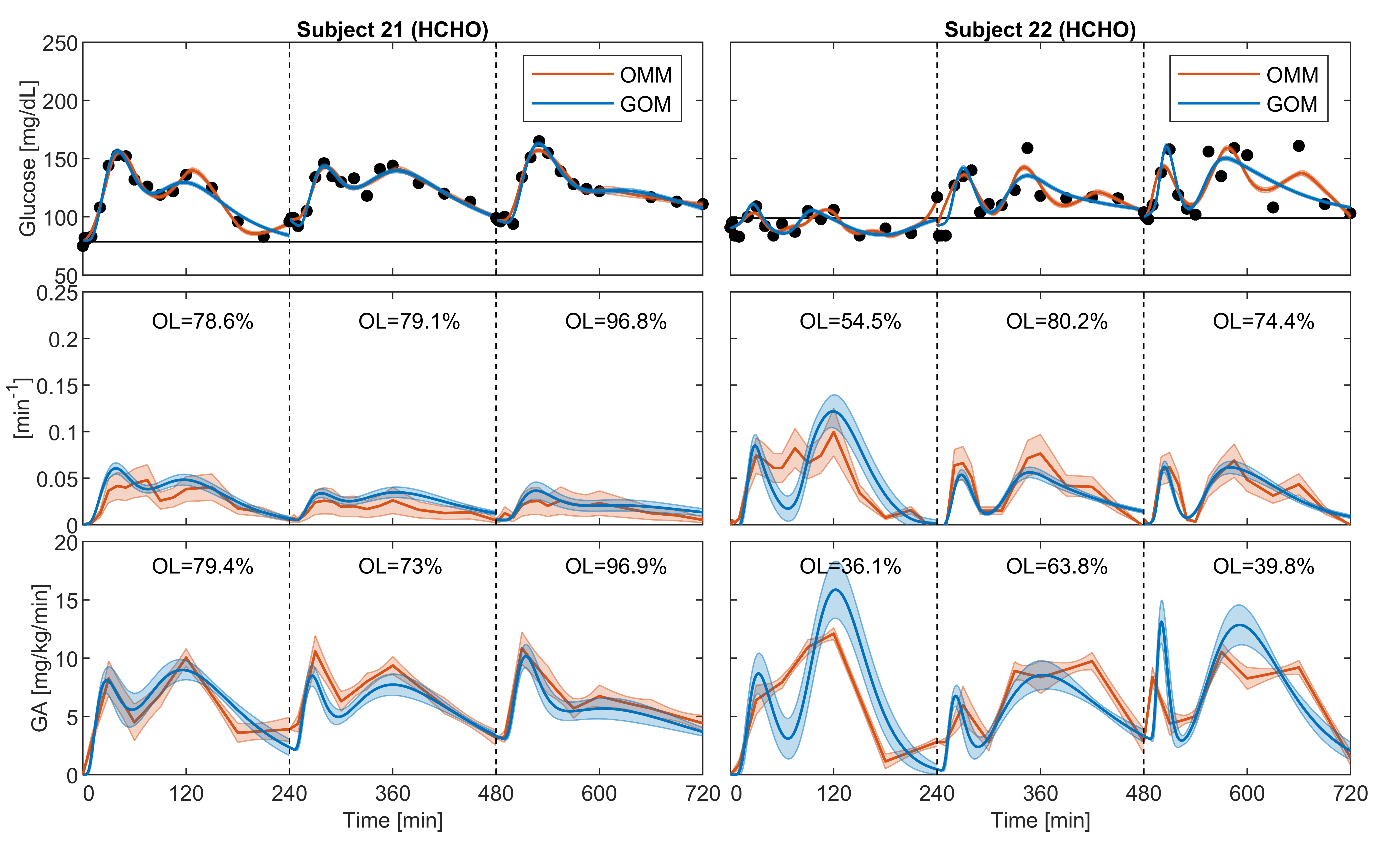


Figure S14: See caption of Figure S4.

## Posterior parameter results

All posterior parameter results in the form of medians and CVs are provided in Figures S15 and S16. The results of parameters $p_{1}$ and $p_{2}$ as well as the GA function parameters are very similar to the corresponding results of the OMM that are already published ^1^.


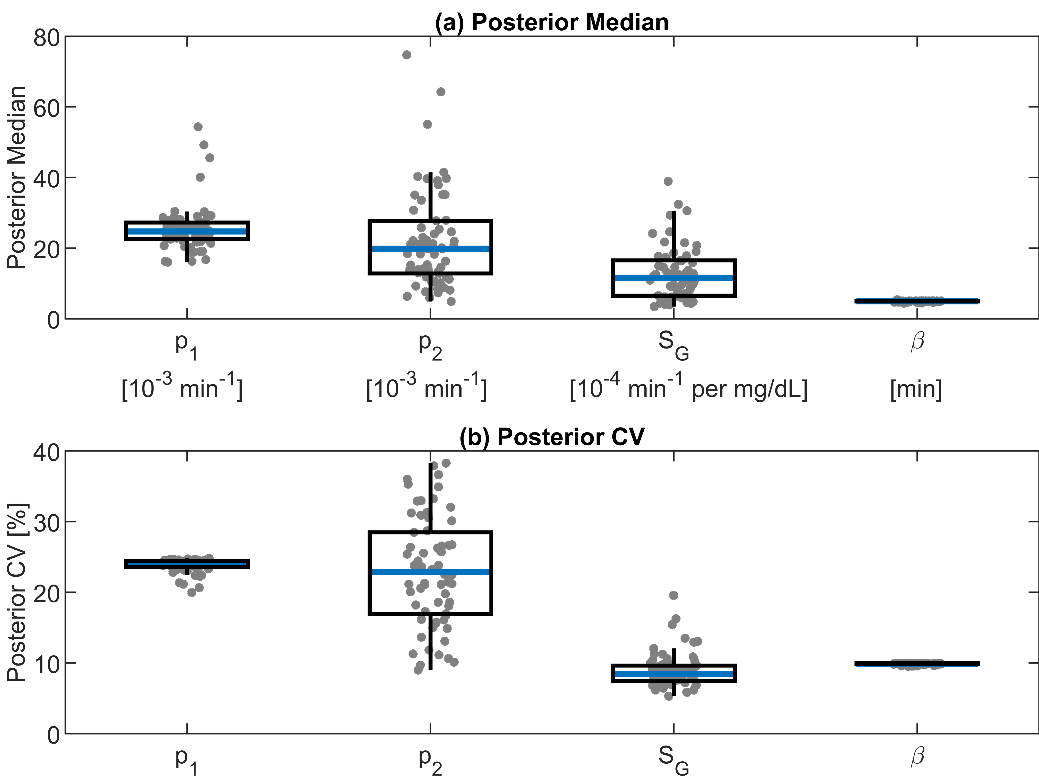


Figure S15: Posterior results of the system parameters. The CVs of the prior distributions are 25, 40, 50 and 10 % for $p_{1}$, $p_{2}$, $S_{G}$ and $\beta$, respectively (see Table S1).


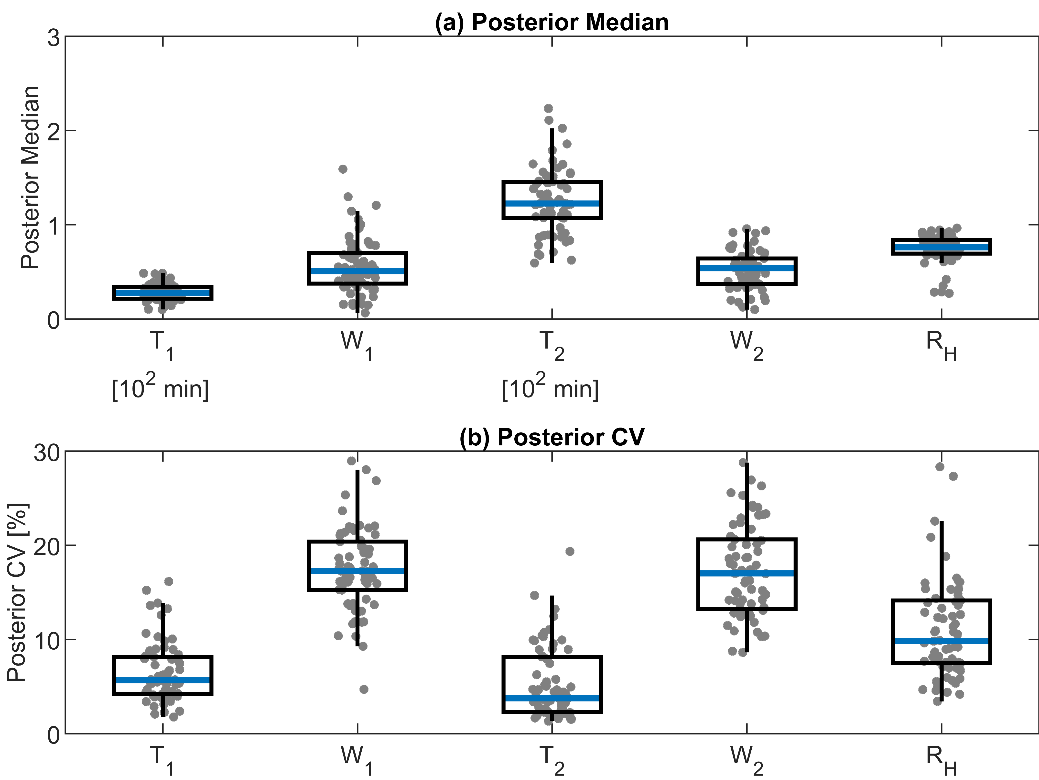


Figure S16: Posterior results of the GA function parameters. All prior distributions have a CV of 30 %

**References**

1. Eichenlaub MM, Hattersley JG, Gannon MC, Nuttall FQ, Khovanova NA. Bayesian parameter estimation in the oral minimal model of glucose dynamics from non-fasting conditions using a new function of glucose appearance. *Comput Methods Programs Biomed*. 2021;200:105911. doi:10.1016/j.cmpb.2020.105911

2. Eichenlaub MM. On the relationship between a Gamma distributed precision parameter and the associated standard deviation in the context of Bayesian parameter inference. *ArXiv210106289 Cs Stat*. Published online January 15, 2021. Accessed February 12, 2021. http://arxiv.org/abs/2101.06289

3. Dalla Man C, Caumo A, Basu R, Rizza R, Toffolo G, Cobelli C. Minimal model estimation of glucose absorption and insulin sensitivity from oral test: validation with a tracer method. *Am J Physiol Endocrinol Metab*. 2004;287(4):E637-43. doi:10.1152/ajpendo.00319.2003
